# Behçet oculaire

**DOI:** 10.11604/pamj.2017.26.237.1175

**Published:** 2017-04-25

**Authors:** Ahmed Alami, Mohamed Kriet, Karim Reda, Abdelkader Laktaoui, Abdelbaare Oubaaz

**Affiliations:** 1 Service d’Ophtalmologie, 3^ème^ Hôpital Militaire de Laâyoune, Maroc; 2 Service d’Ophtalmologie, Hôpital Militaire Avicenne de Marrakech, Maroc; 3 Service d’Ophtalmologie, Hôpital Militaire d’Instruction Mohamed V de Rabat, Maroc; 4 Service d’Ophtalmologie, Hôpital Militaire Moulay Ismail de Meknes, Maroc

**Keywords:** Maladie de Behçet (MB), vascularite, uvéite, fonction visuelle, immunosuppresseurs, Behcet's disease (BD), vasculitis, uveitis, visual function, immunosuppressive

## Abstract

L'objectif était de déterminer les caractéristiques cliniques, thérapeutiques et pronostiques de l'atteinte oculaire chez les patients atteints de la maladie de Behçet, pris en charge dans notre service d'ophtalmologie. Nous présentons une étude rétrospective d'une série de 20 patients colligés à l'hôpital militaire de Laâyoune. Les patients ont bénéficié d'un examen ophtalmologique complet, d'une angiographie à la fluorescéine si nécessaire. Un examen OCT est réalisé chez deux patients. Dix cas d'uvéite antérieure dont un cas s'est compliqué d'hypertonie oculaire; Deux cas d'uvéite intermédiaire; huit cas d'atteinte du segment postérieur dont un cas s'est compliqué d'hémorragie intravitrienne. La maladie de Behçet (MB) est une affection inflammatoire systémique idiopathique actuellement classées au sein des vascularites primitives non nécrosantes. L'atteinte oculaire de la maladie de Behçet est fréquente et grave, mettant en jeu le pronostic visuel des patients. La maladie de Behçet est fréquente au Maroc, engageant le pronostic visuel, rendant la collaboration entre ophtalmologistes et internistes particulièrement importante.

## Abstract (en)

Our study aimed to determine the clinical, therapeutic and prognostic features of ocular involvement in patients with Behçet's disease treated in our Department of ophthalmology. We conducted a retrospective data collection from medical records of 20 patients treated at the military hospital in Laayoune. All patients underwent complete ophthalmological examination and fluorescein angiography if necessary. OCT exam was performed in two patients. Ten patients had anterior uveitis, complicated in one case by ocular hypertonia; two patients had intermediate uveitis; eight patients had posterior segment involvement complicated in one case by intravitreal hemorrhage. Behcet's Disease (BD) is an systemic idiopathic inflammatory disease currently classified within primary non-necrotizing vasculitis. Ocular involvement is common and severe in Behçet's disease, with the potential to compromise the visual prognosis. Behcet's disease is common in Morocco. It can compromise patient’s visual prognosis making the collaboration between ophthalmologists and internists particularly important.

## Introduction

La maladie de Behçet (MB) est une vascularite multisystémique d'étiologie inconnue et dont l'évolution est caractérisée par une alternance de poussées et de rémissions. L'atteinte oculaire représente l'un des critères diagnostiques majeurs de cette affection. Elle présente également un intérêt pronostique et thérapeutique. La cécité en demeure la complication la plus redoutable. L'objectif de notre travail est de préciser les caractéristiques cliniques, thérapeutiques et pronostiques de l'atteinte oculaire chez les patients atteints de MB, pris en charge dans notre service d'ophtalmologie.

## Méthodes

Nous présentons une étude rétrospective d'une série de 20 patients colligés à l'hôpital militaire de Laâyoune. Sont retenus dans cette étude les patients présentant un Behçet oculaire diagnostiqué selon les critères de l'international study group of Behçet disease. Les patients ont bénéficié d'un examen ophtalmologique complet (mesure de l'AV, l'examen à la lampe à fente, l'étude du Fond de l'œil, d'une angiographie à la fluorescéine si nécessaire. Un examen OCT est réalisé chez deux patients.

## Résultats

**Figure 1:**
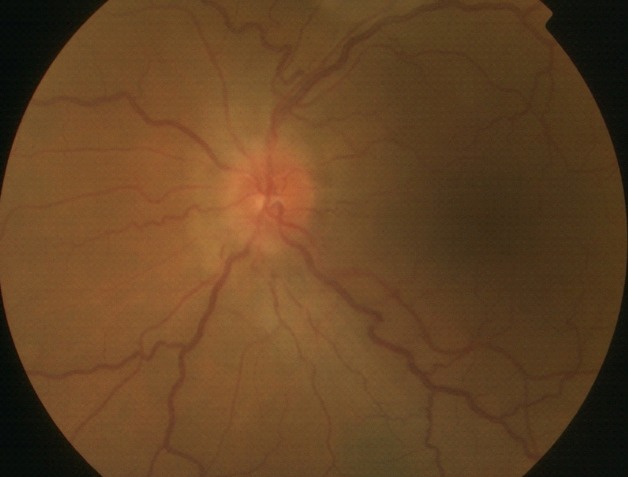
Rétinographie couleur de l’œil gauche montre un œdème papillaire au cours de la maladie de Behçet

**Figure 2:**
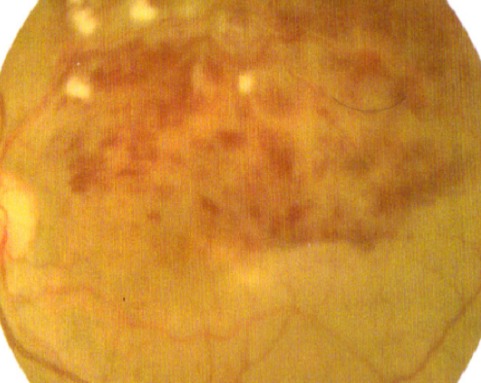
Rétinographie couleur de l’œil gauche montre une occlusion de la veine temporale supérieure secondaire à la maladie de Behçet

**Figure 3:**
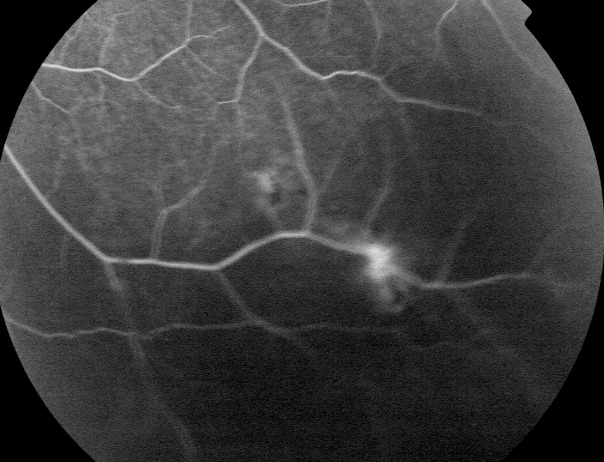
Séquence angiographique met en évidence une vascularite rétinienne au cours de la maladie de Behçet

**Figure 4:**
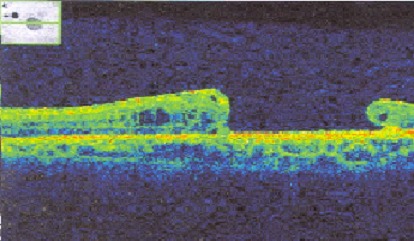
OCT maculaire objective un trou maculaire en pleine épaisseur, compliquant la maladie de Behçet

**Figure 5:**
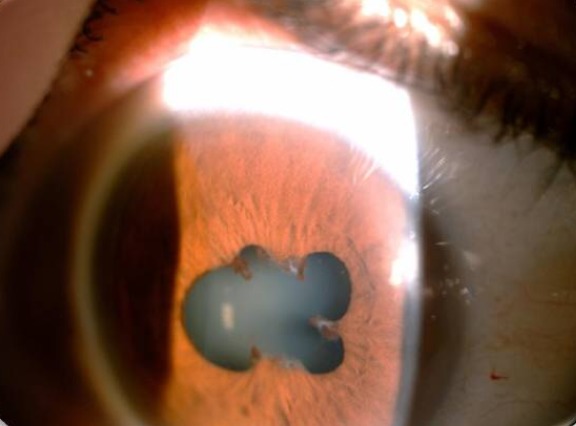
Synéchie irido-cristallinienne compliquée d’hypertonie oculaire au cours de la maladie de Behçet

L'âge moyen de nos patients est de 34 ans, avec une prédominance masculine (14 hommes et 4 femmes). L'acuité visuelle est inférieure à 1/10 chez tous les patients. L'examen à la lampe à fente (LAF) a objectivé Une uvéite antérieure non granulomateuse chez dix patients, dont trois à hypopion; Une uvéite intermédiaire chez deux patients; un œdème papillaire chez un cas (Figure 1); Une vascularite rétinienne avec des périphlébites chez trois patients; une occlusion d'une branche veineuse chez un patient (Figure 2); Un trou maculaire chez deux patients; Une maculopathie chez un patient. Le Bilan biologique montre un Syndrome inflammatoire non spécifique. L'Angiographie à la fluorescéine objectivant la vascularite chez trois cas (Figure 3), l'ischémie maculaire avec élargissement de la zone centrale chez un cas et l'ischémie de la périphérie rétinienne chez un malade. L'OCT montre un trou maculaire de pleine épaisseur chez deux cas (Figure 4). L'échographie oculaire montrant une hémorragie du vitré chez un cas. Le traitement a consisté en Une corticothérapie topique avec des mydriatiques chez dix patients; Un bolus de corticoïde chez trois patients, 10 mg/Kg /Jours de methylprédinésolone puis relais par voie orale 1mg /kg/j à doses dégressives; Injection sous tenoniènne de Triamcinolone chez deux patients; Les immunosuppresseurs à base de l'azathioprine (2mg/kg) chez trois patients; photocoagolation rétinienne chez un malade. L'évolution est marquée par une régression des signes inflammatoires avec remontée de l'acuité visuelle 7/10 chez neuf patients, limitée à 4/10 chez deux patients, 3/10 chez un patient et inferieure à 1/10 chez cinq patients. Un cas d'uvéite antérieure s'est compliqué de synéchies irido-cristallinienne (Figure 5) avec hypertonie aigue ayant nécessitée une iridotomie au laser Yag. L'Hémorragie intravitréenne, suite à une ischémie rétinienne étendue avec formation de néovaisseaux chez un cas, imposant une vitréctomie postérieure avec laser endo-oculaire pour les zones ischémiques.

## Discussion

La maladie de Behçet (MB) est une affection inflammatoire systémique idiopathique caractérisée par une inflammation intraoculaire, des ulcérations orales et génitales, des lésions cutanées ainsi que de nombreuses autres atteintes viscérales [1]. Elle peut concerner quasiment tous les organes et est actuellement classées au sein des vascularites primitives non nécrosantes [2, 3]. Sa prévalence étant maximale dans les pays du bassin méditerranéen et l'Asie et, surtout, en turquie, ou la prévalence est estimée entre 1/10 000 et 1/1 000 habitants [4, 5]. Le sexe masculin est nettement plus touché, en effet nous avons noté une prédominance masculine dans notre série avec un sexe ratio de 3,5. Les mécanismes physiopathologiques évoqués dans cette pathologie sont complexes et font intervenir plusieurs facteurs probablement intriqués : prédispositions génétiques [6, 7], agent infectieux [8, 9], dérégulation du système immunitaire et des médiateurs de l'inflammation [10, 11], protéines de réponse au choc thermique [12], stress oxydatif [13], lipides de peroxydation [14], autres facteurs environnementaux. D'autres facteurs pathogéniques secondaires, incriminés plus directement en rapport avec l'activation des processus immuno-inflammatoires [8, 10]. Le diagnostic de MB est retenu chez nos patients selon les critères du groupe international d'étude sur la MB [15] incluant La présence d'ulcérations buccales récidivantes associées à au moins deux des atteintes suivantes: des ulcérations génitales, une atteinte oculaire inflammatoire, une atteinte cutanée, un pathergy test positif. Tous nos patients présentaient des épisodes d'aphtose buccale. Sur le plan ophtalmologique, les atteintes du segment antérieur sont les plus fréquentes, mais celles du segment postérieur sont plus graves sur le plan de la fonction visuelle [16]. L'atteinte est bilatérale dans la plupart des cas; Dans notre série elle était d'emblé bilatérale chez 50% des cas.

Les atteintes antérieures sont caractérisées par une uvéite antérieure qui constitue l'atteinte la plus fréquente dans notre contexte ; associée dans un tiers des cas environ à un hypopion qui est un signe évocateur, mais il n'est ni pathognomonique ni constant au cours de la MB. L'hypertonie oculaire peut être liée aux synéchies antérieures, à une fermeture de l'angle iridocornéen secondaire à une séclusion pupillaire, ou à la prescription de corticoïdes locaux ou généraux. La cataracte compliquée constitue un obstacle dans la surveillance de l'acuité visuelle et du segment postérieur; dans notre série nous avons retrouvé trois cas de cataracte secondaire à la corticothérapie. Les atteintes postérieures peuvent êtres très variées [16, 17], rétiniennes (œdème, hémorragie, exsudats, neovaisseaux pré-rétiniens), papillaire (hyperhémie, neovascularisation, 'dème papillaire), vitréennes( hyalite, DPV, hémorragie), vascularites rétiniennes ( engainement artériel ou veineux, dilatation veineuse, occlusion veineuse), maculaire (œdème maculaire cystoïde, nèovascularisation sous-rétinienne, membrane epi-rétinienne, et même trous maculaires bien que ces derniers soient très rares [18, 19], hémorragies, exsudat, ischémie [20, 21]). Dans le cas des vascularites rétiniennes, les infiltrats rétiniens et les occlusions de branches veineuses rétiniennes sont assez caractéristiques de la MB [22]. La vascularite oblitérante nécrosante est caractéristique de l'infection et constitue un élément pronostique important avec souvent une baisse d'acuité visuelle à terme [16]. La perte totale de l'acuité visuelle survient en moyenne 3,36 ans après la survenus des premières signes ophtalmologiques [23]. Dans notre étude on a eu cinq patients dont l'acuité visuelle se limitait au compté des doigts. Le traitement de la MB est actuellement purement symptomatique et vise à juguler les poussées et à atténuer les séquelles. Les corticoïdes et les immunosuppresseurs ont longtemps été la base du traitement. Les corticoïdes ont une action anti-inflammatoire immédiate sur les poussées aigues, mais ils doivent être renforcés par des immunosuppresseurs. Le traitement de l'atteinte oculaire de la MB est encore non codifié, chaque atteinte visuelle nécessite un traitement propre, indépendant du traitement de fond. L'utilisation plus rationnelle et plus extensive des immunosuppresseurs explique probablement en partie l'amélioration du pronostic visuel entre les années 80 et 90. Le risque de rechute après arrêt total ou ponctuel des traitements est possible. La prise en charge des complications oculaires est également importante pour améliorer le pronostic visuel des patients: chirurgie de la cataracte et du glaucome, traitement au laser des ischémies rétiniennes, vitrectomie postérieure en cas d'hémorragie intravitrienne ou trou maculaire. L'atteinte oculaire de la MB est fréquente et grave, mettant en jeu le pronostic visuel des patients, en effet, l'uvéite de la MB est responsable d'un grand nombre de cécité ou de basse vision dans les pays où la maladie est la plus fréquente. Le pronostic dépend également de la rapidité et de la précocité de la prise en charge rendant la collaboration entre ophtalmologistes et internistes particulièrement importante.

## Conclusion

La maladie de Behçet est fréquente au Maroc. Touche le sujet jeune de sexe masculin. Le pronostic oculaire de la MB est amélioré par une prise en charge précoce et un suivi clinique et angiographique rigoureux. La collaboration étroite entre ophtalmologistes et internistes est donc particulièrement importante afin de préserver l'avenir visuel des patients.

### Etat des connaissances actuelle sur le sujet

- La maladie de Behçet (MB) est une vascularite multisystémique d'étiologie inconnue, dont sa prévalence étant maximale dans les pays du bassin méditerranéen et l'Asie;
- L'atteinte oculaire représente l'un des critères diagnostiques majeurs de cette affection.

### Contribution de notre étude à la connaissance

- Rapporter l'expérience de notre service sur les aspects: épidémiologique, clinique, thérapeutique et évolutif de l'atteinte oculaire par la maladie de Behçet;
- Rappeler que la maladie de Behçet engage le pronostic visuel et insister sur l'importance de la collaboration entre l'ophtalmologiste et l'interniste pour une meilleur prise en charge de cette affection multisystémique.
